# A Usability Study of Physiological Measurement in School Using Wearable Sensors

**DOI:** 10.3390/s20185380

**Published:** 2020-09-20

**Authors:** Nattapong Thammasan, Ivo V. Stuldreher, Elisabeth Schreuders, Matteo Giletta, Anne-Marie Brouwer

**Affiliations:** 1 Human Media Interaction, Faculty of Electrical Engineering, Mathematics and Computer Science, University of Twente, 7522 NB Enschede, The Netherlands; ivo.stuldreher@tno.nl; 2 Perceptual and Cognitive Systems, The Netherlands Organisation for Applied Scientific Research (TNO), 3769 DE Soesterberg, The Netherlands; anne-marie.brouwer@tno.nl; 3 Department Developmental Psychology, Institute of Psychology, Tilburg University, 5000 LE Tilburg, The Netherlands; e.schreuders@uvt.nl (E.S.); matteo.giletta@ugent.be (M.G.); 4 Department of Developmental, Personality and Social Psychology, Faculty of Psychology and Educational Sciences, Ghent University, 9000 Ghent, Belgium

**Keywords:** wearables, EDA, heart rate, peripheral physiology, ambulatory monitoring

## Abstract

Measuring psychophysiological signals of adolescents using unobtrusive wearable sensors may contribute to understanding the development of emotional disorders. This study investigated the feasibility of measuring high quality physiological data and examined the validity of signal processing in a school setting. Among 86 adolescents, a total of more than 410 h of electrodermal activity (EDA) data were recorded using a wrist-worn sensor with gelled electrodes and over 370 h of heart rate data were recorded using a chest-strap sensor. The results support the feasibility of monitoring physiological signals at school. We describe specific challenges and provide recommendations for signal analysis, including dealing with invalid signals due to loose sensors, and quantization noise that can be caused by limitations in analog-to-digital conversion in wearable devices and be mistaken as physiological responses. Importantly, our results show that using toolboxes for automatic signal preprocessing, decomposition, and artifact detection with default parameters while neglecting differences between devices and measurement contexts yield misleading results. Time courses of students’ physiological signals throughout the course of a class were found to be clearer after applying our proposed preprocessing steps.

## 1. Introduction

There is a long history in psychological science to assess psychological and emotional processes using physiological measurements. For example, peripheral measures such as electrodermal activity (EDA) and heart rate (HR) have been widely used as indicators of emotional valence and arousal [1], mental workload [2], stress, decision making [3], and engagement [4] in laboratory settings. Recent technical advancements in the development of unobtrusive wearable sensors to assess EDA and HR offer the opportunity to monitor physiological processes under ecological circumstances in people’ daily lives. In addition to other measurement tools such as questionnaires and observation, wearable physiological sensors provide unique, detailed information about individuals’ cognitive and emotional states [5]. They can provide information about mechanisms underlying psychological processes, they are non- or minimally invasive and require no attention of users, thereby allowing continuous assessment. The past decades have witnessed an increased popularity in deploying wearable sensors to record psychophysiological signals in real life.

However, measuring physiological signals in a real-life setting comes with a number of challenges. First, unlike a controlled laboratory setting where the signal quality is high, signals recorded from ambulatory settings can be noisy due to excessive body movement, electrode disturbance, or limitations of the device. Notably, physiological signals are often confounded with factors like these [6], therefore, threatening the validity of ambulatory assessments. Second, deploying wearable physiological sensors in the field requires nuanced understanding of the cost and benefit involved [7], device acceptability, usability, and robustness [8]. Finally, while there is a call for the validation of commercially available wearable physiological sensors by independent parties, only a few of them had been validated. For example, only five percent of HR wearable technologies have been formally validated against gold-standard measurement [9], and only ten percent have been developed as research-grade technologies appropriate for the recording in the wild [9]. More systematic, transparent, and rigorous evaluations are necessary prior to adopting the measurement of real-life physiological signals to obtain information about mental processes out of the lab.

Physiological processes can be examined in real life in different populations and across a variety of contexts. Yet, adolescents represent a particularly interesting population, given that during this developmental period, on average, youth are exposed to increased (social) stressors and show heightened emotional and physiological responses to stress as compared to both children and adults [10,11,12,13]. Examining physiological processes as they unfold in adolescents’ daily lives may therefore contribute to a better understanding of the mechanisms underlying the development of internalizing symptoms and emotional disorders, many of which often have their onset in adolescence (e.g., depression and anxiety) [14].

Notably, ambulatory assessment among adolescents can be carried out in the school setting, which represents an ecologically valid environment where the vast majority of adolescents spent most of their time. At school, adolescents are often involved in many cognitively and emotionally salient activities, for example when learning or interacting with their peers, making the school environment a highly appropriate setting to examine psychophysiological processes. Moreover, the school context offers the opportunity to measure in a semi-structured fashion where in contrast to other, “fully wild” scenarios, certain factors are known (e.g., the class schedule). For the current study, we therefore chose to measure physiological signals of adolescents under minimally controlled conditions in a school context.

While some research has been done on students’ engagement [15,16,17,18,19] and emotional states [20,21] using wearable EDA and HR sensors during class, we aimed to extend the existing literature by elaborating on the usability and practical limitations which were often unreported in the literature. Specifically, we aimed to investigate the usability and identify practical issues of physiological signal acquisition using wearable sensors among adolescents in a school context and also to examine the validity of signal processing in this ambulatory setting. Furthermore, we focus on EDA since there are few studies recording EDA in real life compared to HR. This is probably due to the fact that wearables for recording EDA are relatively costly and have not been around as long as HR wearables. In addition, the definition of unrealistic values as well as informative variables is arguably less straightforward. We recorded signals of EDA and HR of 86 students in two different schools. The devices used in this research were a wrist-worn device (to record EDA) and a chest-strap sensor (to record HR), which were reported to be wearable sensor types that receive high levels of acceptance and compliance among children [22]. In this paper, we discuss and present:

- the usability and efficiency of the devices when recording at school; we evaluate the practical feasibility of monitoring physiological signals using wearable sensors in school, where students were allowed to move freely without the instruction to perform any specific task. Handling of devices, recording interference, frequency, and amount of missing data were assessed to quantify the feasibility to measure physiological signals in the field at school.
- signal processing issues in real-life recording; we examined how to deal with invalid EDA signals due to loose sensors, and we evaluated the adequacy of using automatic EDA toolboxes to detect artifacts. This forms the main part of the study. Particularly, this part will be relevant to a broader range of real-life settings than only the school.
- the general pattern of EDA and HR in a classroom setting; to fill the gap between laboratory experiments in an education context and real-world measurement with unrestricted movement and unknown contexts, we chose to focus on the class lectures that can be assumed not to involve vigorous physical activities. We examined time courses of physiological signals of students from start to end of a class lecture.

We collected in total over 410 h of EDA data and 370 h of HR data among 86 adolescents, which is substantially more than the other three studies we are aware of that monitored students using wearable EDA sensors in school [18] and other studies using HR sensors [23]. Although in this study, we focused on the school environment as a relevant exemplary case to monitor physiological activity in real-life, the implications of this work may extend beyond the school setting, to other real-world contexts in which physiological signals are measured using unobtrusive wearable sensors.

## 2. Methods

### 2.1. Participants

The participants were 86 adolescents, enrolled in two secondary schools in The Netherlands. This study was part of a larger longitudinal study examining adolescents’ socio-emotional well-being from a biopsychosocial perspective (Peer Power UP!). The study was approved by the institutional ethics research board of Tilburg University and by participating schools. During this wave of the longitudinal study, several questionnaires were included to explore social and emotional experience during the school week, wherein one particular day involved monitoring physiological signals from the start to the end of the school day.

Participants were all third-year high school students, aged between 14–16 years, with a mean of 14.9 years (standard deviation (SD) = 0.5 years, 51.2% girls). The students were briefed on the study procedures by the research staff one week before the measurement week. An active assent was obtained from the student, and one parent of the child (in case the child was younger than 16 years old) was asked to give consent for participation in the study. Participants received a gift card to thank them for participating in the study.

The day of physiological measurement in the week of study was chosen on the basis of class context and duration of adolescents’ presence at school. In particular, days with gym classes were avoided and the chosen day was preferably the day that students spent the longest time at school in the week. As a result, physiological activity was recorded in adolescents from 17 different classes with different sizes on seven different days in the spring of 2019.

### 2.2. Sensors and Procedure

**Figure 1:**
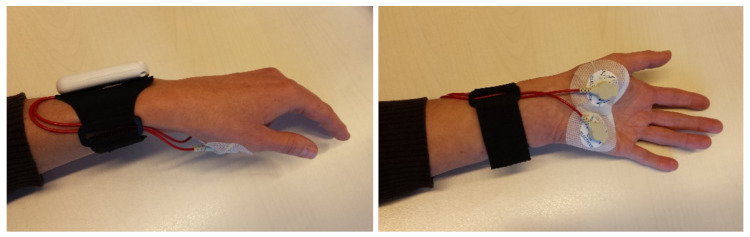
Electrodermal activity (EDA) sensor used in the experiment (EdaMove 4).

EDA signals were recorded through a wearable, unobtrusive, and non-invasive wristband, EdaMove 4 (Movisens GmbH, Karlsruhe, Germany) [24], worn at the wrist of the non-dominant hand of the participant, with two solid gelled Ag/AgCl electrodes (MTG IMIELLA electrode, MTG Medizintechnik, Lugau, Germany, W55 SG, textured fleece electrodes, 55 mm diameter) recording signals from the palmar surface of the hand (Figure 1). Palm-based EDA devices were found to be more sensitive to change of EDA compared to devices measuring at locations on the wrist due to a higher density of sweat glands [25]. The electrodes’ hydrogel layer produces high signal quality by effectively lowering the impedance that exists at the electrode–skin interface. These electrodes have been shown to provide a more accurate biosignal waveform than carbon or stainless steel electrodes [26], and are relatively robust to variations in contact during motion, which is important for recording in an ambulatory setting. In addition, skin-friendly adhesive fleece tape was applied to ensure the attachment of the electrodes over the long period of recording. Adhesive tape was preferred over wearing thin gloves because in pilot recordings these were found to generate discomfort when recording for a long time, affect EDA level by inducing heat at the palm, and sometimes induce artifacts. The EdaMove 4 applied a constant direct current (DC) voltage of 0.5 V to the skin to acquire skin conductance. EDA was measured at a sampling rate of 32 Hz with an input range of 2–100 μS and with a resolution of 14 bits [27]. The device also contains a three-axis accelerometer to capture movement at a sampling rate of 64 Hz and is waterproof [27].

HR was recorded through the Wahoo-Tickr (Wahoo Fitness, Atlanta, GA, USA), a commercially available HR monitoring device, directly worn on the chest. The device reports heart rate in beats per minute (bpm), approximately one time per second, at a resolution of 1 bpm. It does not provide raw inter-beat intervals or measures of heart rate variability. Despite featuring embedded dry electrodes for recording, conductive gel was applied on the recording surface before wearing to improve the conductivity with the skin as the device requires a stable electrode-skin contact for activation. The serial HR data was streamed via Bluetooth communication to an Android mobile phone, which each participant was asked to carry throughout the recording. The streamed data was captured using the Wahoo Fitness Workout Tracker application (Version 1.13.0.94) which displays HR in a real-time fashion. A password was used to prevent participants from interacting with the phone or interfering with the recording.

Every day, before device distribution, time synchronization to an online global clock was performed to ensure the time-alignment across multiple devices. Each device was tagged with a unique numeric label to allow matching of recorded data with the corresponding participant. To equip the participants with the devices in time before school started, participants were instructed to come to school 20 min before the beginning of their first classes. To further motivate participants to be on time, they were rewarded with a 2.50-euro lunch voucher to spend in their school’s cafeteria. All but one of the participants showed up in time. Participants could mostly be instructed and equipped timely by a group of three to five experimenters, some of whom remained at the school until the end of the day. The participants were instructed to refrain from interfering with the sensors and to come to the experimenter whenever encountering problems with sensor attachment. Participants were asked to return the recording devices to the experimenter right after the last class of the day and to report any problems or discomfort encountered during the measurement.

### 2.3. Analysis

After transferring EDA and HR data from the devices to a computer, data from both devices were aligned at one-second resolution, and then preprocessed. In the sections up until Section 3.2, all data, starting at the onset of the recording up until handing in the device, are considered. It includes data recorded during classes as well as during breaks and changing classes. In Section 3.2, we zoom in on data recorded during classes. Details and justification of preprocessing protocol are explained and discussed in Section 3.3.

EDA signals result from the superposition of two different components: skin conductance level (SCL) and skin conductance response (SCR; also referred to as phasic response) [28]. SCRs are short-lasting rises from an initial level in EDA and can be elicited by distinct, arousing events. They represent the most salient characteristic of EDA resulting from the firing of sudomotor nerves that are part of the sympathetic nervous system [29]. The long-lasting slow drift occurring in the absence of obvious stimuli is referred to as SCL or tonic component. It is common to evaluate phasic and tonic EDA components separately. Recently, studies have proposed a variety of methods to decompose EDA signals into its two components. One of the commonly used techniques makes use of a deconvolution operation [30] to extract phasic activity from the raw EDA signal and has been proven to be a robust approach capable of revealing stress response in EDA recorded from lab-grade equipment [31]. This approach has been made easily accessible in the freely available and widely used toolbox Ledalab [30]. It extracts a range of psychophysiological relevant SCR variables from the EDA signal, including rising time, amplitude, and frequency of peak occurrence. We here used this toolbox to decompose our cleaned EDA signals into phasic and tonic components.

Following a previous study on EDA-based stress detection [32], we extracted the following features from EDA signals within a specific time window.

- Raw EDA signal: mean and median.
- Phasic component: mean and median.
- Tonic component: mean and median.
- Peak amplitude: mean and median; peaks were detected by Ledalab defined as the point where underlying EDA “driver” slope changes its sign.
- Number of peaks: number of all peaks, number of peaks with amplitude higher than 0.05 μS, number of peaks with amplitude higher than 1 μS

To smooth the stair-case trace in HR signal caused by quantization error, we applied a moving-average filter to preprocess the signal with a window size of four seconds, as recommended in another study using the same HR sensor [33]. For accelerometer signals captured by EdaMove, we calculated the root mean square of three axes of the accelerometer to derive a single time-series of device acceleration, hereafter referred to as accelerometer data. Similar as for the EDA data, we extracted the mean and median from HR signals and accelerometer data in specific time windows.

## 3. Results

### 3.1. Usability

#### 3.1.1. Participants’ Handling of the Devices

Although the measurement was done under uncontrolled recording circumstances and without direct supervision, all devices were returned properly without any damage. The presence of an experimenter was found important and effective for problems with sensor fixation and ensuring measurement progress. EDA signals were stored locally and could not be viewed or interfered with by participants. However, a few participants managed to read the history of the HR measurement by pausing the recording and visualizing data via the application on the phone. In these rare cases, this led to the transient loss of HR signal, usually occurring at the beginning of the day. We also observed strongly increased HR by intentional intensive physical activities of one of these participants wishing to see HR change.

#### 3.1.2. First Impression: Missing Data and Overall Data Quality

For EDA data, we encountered a technical problem during file manipulation resulting in the loss of one participant’s data. Early visual inspection of raw EDA data showed that spiky artifact patterns in EDA, frequently reported in previous ambulatory recordings using dry electrodes, were not prominent in our study, suggesting the superiority of Ag/AgCl hydrogel electrode in term of the robustness to movements.

For six out of 86 participants, we encountered a technical problem when exporting the HR data and during file manipulation, resulting in the loss of all HR data from these participants. Intermittent data loss in HR recordings can happen due to either the failed connection with the phone or electrode–skin contact loss. Missing data were reported to be as high as 8% in a prior study [34] recording HR in-situ using chest sensors. In our case, we lost 3.30% of HR data in the remaining 80 participants. Averaged HR loss per participants was low (mean = 2.96%, median = 0.87%, SD = 6.79%). We think that the most important factor contributing to the low loss rate is caused by the application of gel, which seemed to have successfully maintained the contact between HR electrode and skin.

**Figure 2:**
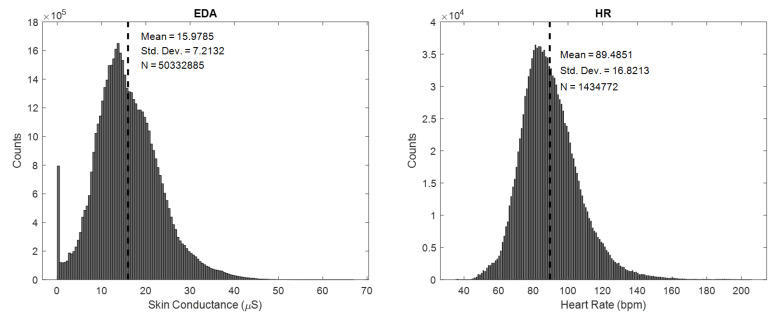
Distribution of EDA and heart rate (HR) signals in the bin width of 0.5 μS and 0.5 bpm, respectively.

Figure 2 shows the amplitude of all raw EDA and HR samples aggregated from all participants included in the analyses. Participants’ recorded HR was on average 89.49 (SD = 16.82) bpm. Most of HR samples fall between resting-state HR of European adolescents (77.4 and 81.1 bpm for boys and girls, respectively [35]) and HR during moderate movement (97 and 101 bpm for boys and girls, respectively calculated by averaging between standing rest-state HR and the lowest exercising HR [36]). The EDA of participants (mean = 15.98, SD = 7.21 μS) was considerably higher than found using wearable EDA devices recording at the wrist with dry electrodes [7,37] which can be explained by the higher density of sweat glands at the palm [25]. The EDA panel Figure 2 shows a conspicuous peak on the left reflecting a high number of EDA signals lower than 0.5 μS. The is likely caused by the loss of attachment between electrode and palmar skin. This part of the data should be removed as discussed in the next section.

### 3.2. Signal Processing of Real Life Eda

#### 3.2.1. Signal-Loss Due to Loose Sensor

Stability of the electrode attachment to the skin is a major issue in an ambulatory recording. Electrode adhesiveness with the skin can decline because of oil and perspiration [38] that might vary during the day. Intermittent physical disconnection between electrodes and the skin leads to a sudden drop and rise in EDA signal [39,40]. Kleckner and colleagues suggested that abrupt drops below 0.05 μS, which is the generally accepted minimal amplitude criterion of SCR [25], are likely caused by signal loss due to the electrode detachment when recording from wrist-worn dry-electrode EDA sensor and should be discarded from the analysis [41]. Doberenz and colleagues suggested a threshold of 0.5 μS [42] when recording EDA from fingers using gelled electrodes. Based on the visual inspection of our data, we chose to set the minimal threshold of raw EDA signals to be considered as possibly valid to 1 μS.

Data surrounding signal-loss portions reflect the transition from valid to invalid portion and vice versa, rather than conveying psychophysiological information. These transitional portions should therefore also be discarded. Kleckner and colleagues recommend removing 5-s portions before and after the signal-loss period [41]. A motivation behind this transitional period size was not given, but when determining this size, there is always a compromise between information loss and the assurance that the transition effect is removed. We here varied the size of the transitional period and investigated the effect on signal quality. When signal-loss periods happen quickly after each other, e.g., because participants became aware of the low adhesiveness of electrode and unsuccessfully re-attached the electrode by pressing, the signal between consecutive signal-loss periods may be unreliable. Apart from this, too short epochs will be unable to capture a psychophysiological response given its time course over several seconds [43]. Therefore, the portion between signal-loss periods (referred to hereafter as an inter-loss period) should be removed if the length does not satisfy a given minimal requirement, which we also investigated here.

To examine the effect of the parameter settings for data removal on signal quality, we varied the size of the transitional period from 0 to 2 s with 0.25-s incremental steps, and the inter-loss period from 0 to 6 s with 0.5-s incremental steps. In order to validate the effect of the different combinations of settings, we calculated metrics from the processed signal that reflect successful removal of invalid data. These metrics are defined in the first four rows of Table 1. A valid signal should show few jumps, few excessively steep slopes (spikes in EDA signal that have much steeper slopes compared to genuine SCR [25]), few short valid epochs, and low entropy (a complex signal with highly oscillating waveform as may be caused by abrupt descent and ascent traces of EDA, exhibits higher entropy, i.e., requires more bits to encode information). In addition, it is important to not lose too much data, i.e., not to remove too much data around sensor loosening events (definition in the last row of Table 1).

To combine the scores from different metrics in one “signal loss artifact score”, we normalize values into the range [0, 1] using a minimum–maximum approach. For the metrics that involve counting (C1, C2), normalization was done across removal settings within-participant first, and the means of normalized scores were then obtained from all participants. Meanwhile, other metrics (M1, M2, M3) were zero-mean adjusted for each participant and averaged across participants. Then, normalization was done across removal settings.

**Figure 3:**
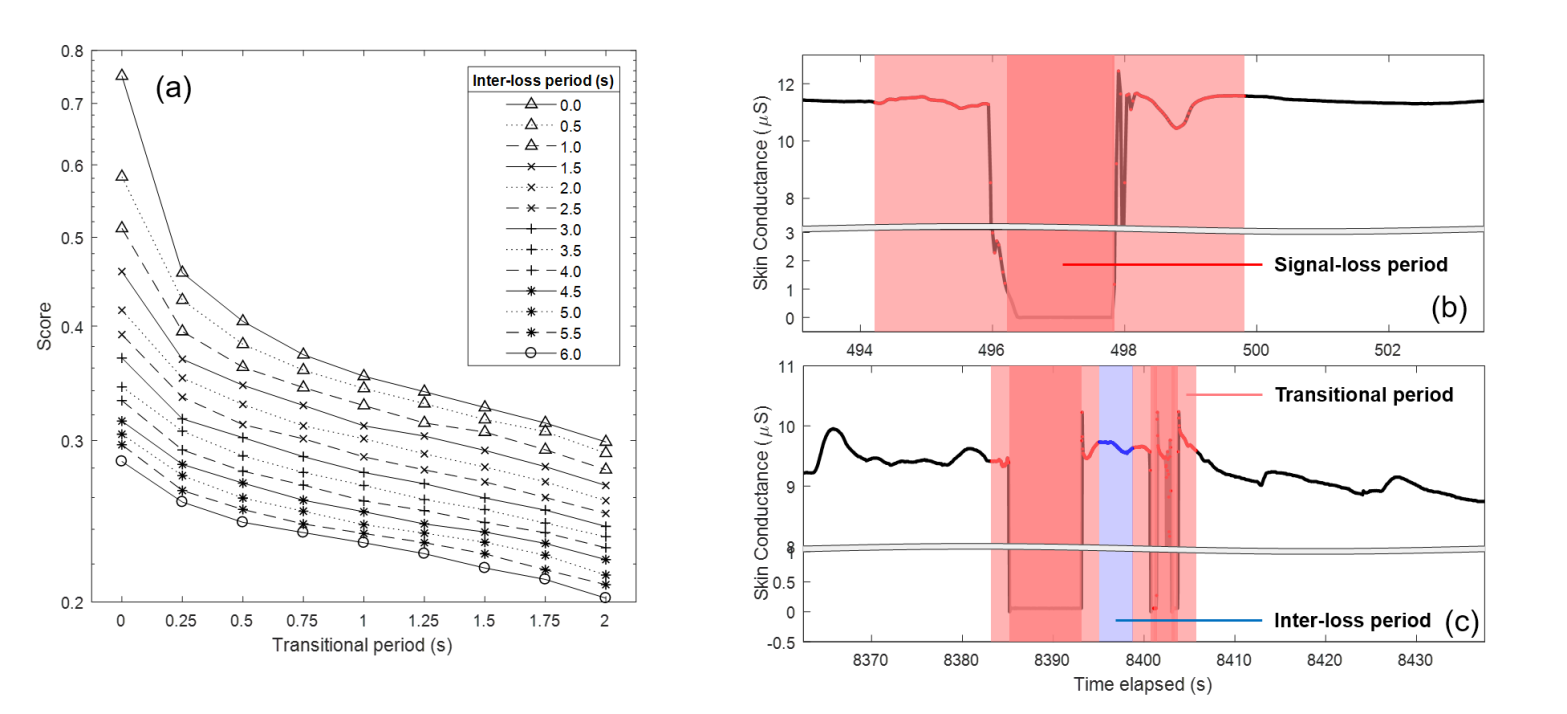
Results on the removal of transitional and inter-loss period. The effect of variations of the transitional and inter-loss period on the averaged normalized metrics is shown in (**a**). Examples of data portions that were marked as invalid periods are shown in (**b**,**c**).

The effect of variations of the transitional and inter-loss period on the averaged normalized metrics is shown in Figure 3a. Note that the vertical axis is on a logarithmic scale. The sharp decrement of the artifact score when expanding the transitional period from 0 to 0.25 s indicates the necessity to consider a transitional period when cleaning the EDA signal. Further increasing the size of the transitional period steadily lowers the score. In addition, the removal of the inter-loss period plays a role in decreasing the scores, especially when the transitional period is zero. When considering both, the lowest score was obtained when maximizing transitional period to two seconds and inter-loss period to six seconds, which is also in line with the recommended minimum length of 6 s to accurately measure the SCR [45]. An example of data portion after removal invalid periods is illustrated in Figure 3b. It should be noted that the weight of each metric can be adjusted based on the variable importance given to the metric depending on the measurement context. For instance, if data loss is more critical, one can consider adjusting the weight for the M3 before averaging.

As a result of removing EDA data below 1 μS, a transitional period of two seconds and an inter-loss period of six seconds, we lost around 5.80% of data. In 11 participants whose EDA signals were heavily contaminated by artifacts, we discard on average 29.41% (SD = 14.62%). However, for the other 74 participants, the discarded data is below 10% (mean = 1.78%, SD = 2.65%), which is acceptable for the recording in an ambulatory setting.

The duration of signal-loss period strongly varied across participants. The short duration of signal-loss period (grand median = 0.406, grand mean = 0.959, SD = 1.889 s, calculated from median of each participant) suggests that the large portion of signal-loss period was rather caused by frequent short occurrences of electrode interference rather than few lengthy periods of disconnected electrodes. The occurrence of signal-loss period also highly varied across participants; median occurrence is 28 times, while average occurrence is 155.78 times with SD = 377.74 times.

*Points of attention and recommendations for Section 3.2.1*: The removal of EDA data that has ultra-low values due to loose sensors is not trivial but received little attention in ambulatory physiological measurement research. Overlooking the signal-loss period but directly applying classical filtering or data smoothing techniques might attenuate sharp drops in the EDA trace. As a result, the EDA in the signal-loss period may surpass the minimal allowable range of EDA, and then setting the minimum threshold to discard the signal-loss portion may not work effectively. Therefore, the order of signal preprocessing is a crucial factor. We recommend to discard ultra-low values, discard transitional period surrounding them, and then remove the short-portion between the discarded portions, before applying any filter as discussed in the next section.

#### 3.2.2. Quantization Error and Its Effect on Extracting Scr

Ledalab software performs continuous decomposition analysis (CDA-Ledalab) to decompose signals into phasic response and tonic level [30]. It is one of the most used tools to detect and characterize SCRs. However, the feasibility of applying this toolbox to signals from wearable devices is not intensively studied [46].

A prior validation study [47] reported that EDA signals recorded from EdaMove 4 and a lab-grade EDA sensor looked very similar. However, if we examine the finer details of the EdaMove 4 signal, we found ubiquitous high-frequency noise, especially at the descending curve of EDA during the recovering process following EDA peaks. This type of noise is referred to as quantization noise [48,49] and is caused by limitations of a device in analog-to-digital conversion. The quantization noise can lead to the false detection of SCRs.

Quantization noise in wearable EDA had been previously reported [50,51,52] but gained little attention. A possible solution to deal with this would be to remove all high-frequency noise by using a moving average or by applying a low-pass filter on the EDA signal, as has been recommended in earlier works [32,40], before extracting SCRs using Ledalab. However, there is no consensus on the smoothing parameter (window size), filter type (finite impulse response (FIR)/infinite impulse response), order (2nd- to 32nd-order), and cutoff frequency (0.4–3 Hz) [46]. It is important to realize that the various filters will not only suppress the high-frequency noise but also reduce the amplitude of SCR in lower frequency ranges. In order to elucidate the effect of different filter settings with a special focus on removing quantization noise while keeping the remainder of the signal intact, we deployed a publicly-available dataset. This dataset was collected in the context of a neurophysiological study [4], conducted in a laboratory setting where participants were sitting still for about an hour while listening to an auditory stimulus. EDA was simultaneously recorded by the same device as used in our study, EdaMove 4, as well as by a lab-grade system (ActiveTwo Mk II system; BioSemi, Amsterdam, The Netherlands). The latter system recorded EDA, with a sampling rate of 1024 Hz, using two gelled electrodes placed on the ventral side of middle and index finger of the participants. We here use this dataset to examine the effect of filters and their settings on extracting SCRs from the EdaMove data using Ledalab. Data from the ActiveTwo system served as gold standard.

**Figure 4:**
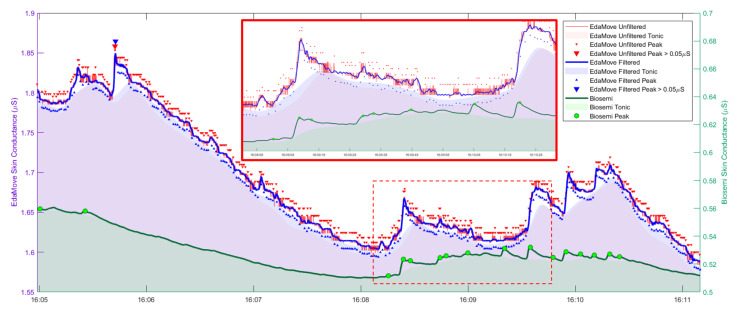
Continuous decomposition analysis (CDA-Ledalab) results from EdaMove signals before and after filtering and from ground-truth ActiveTwo signal. The smaller box illustrates in finer details. Locations of the estimated phasic peaks are shown. Significant peaks with the estimated amplitude higher than 0.05 μS are highlighted by using larger marks. Note that the scale on the left-vertical axis is for EdaMove signals, while the scale on the right-vertical axis is for ActiveTwo signal.

Many small peaks were detected in the EdaMove signal by CDA-Ledalab when no peaks were detected from ActiveTwo signal, as shown in Figure 4, demonstrating the adverse effect of quantization noise. Applying a hard low-pass filter (32nd order) does not significantly reduce the number of detected small peaks as the tiny oscillations due to quantization noise still remain. Setting a criterion in Ledalab of peak height to be 0.05 μS [25] excessively reduced the number of peaks to only one detected peak, as opposed to 16 peaks detected from ActiveTwo signal, in the exemplified data. A plausible reason is that the noise-driven oscillation at the summit of SCR forced CDA-Ledalab to decompose the phasic component into multiple sub-SCRs with smaller amplitudes. Therefore, setting a firm threshold to discard smaller peaks without alleviating quantization noise can lead to the issue of missing information.

**Figure 5:**
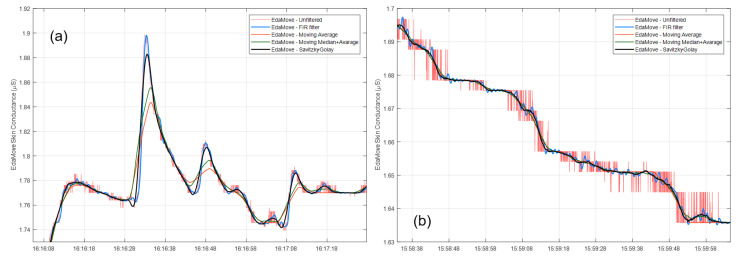
Comparison of EdaMove signals before and after applying various filtering/smoothing techniques at two different time periods (**a**,**b**).

We further tested increasing the order of FIR filter, equivalent to enlarging the size of the moving-average filter, to obtain a smoother curve. However, this leads to decreased amplitudes of SCR, as demonstrated in Figure 5a. This is undesirable because peak amplitude is an important feature in psychophysiological research [29,31]. Recently, empirical mode decomposition has been proposed to be superior to the moving average approach to remove quantization noise in EDA. However, despite yielding higher low-frequency-to-high-frequency ratio compared to the moving average approach, empirical mode decomposition was observed to strongly decrease peak amplitude and significantly lower number of detected peaks [52]. It is difficult for the classical filtering technique to alleviate the issue of quantization noise while preserving the amplitude height of SCRs. Ledalab offers data smoothing options to users including the Hanning window, moving average, and Gaussian method. However, these smoothing techniques are similar to the ones described above, with the same associated disadvantages.

Instead of using a linear filter, we propose to use Savitzky–Golay (S-G) filtering [53], which is a data smoothing technique that has the advantage of preserving amplitude of peaks. An S-G filter is an FIR filter that smooths data by fitting a signal using a local least-squares polynomial approximation, without much data distortion. By using a low-degree polynomial, the window size can be as large as multiple seconds while still preserving SCR amplitude. S-G filters have been used for denoising various biomedical signals, including electrocardiographic data [54]. It was recently used to remedy quantization error in the video domain [55], which suggested its potential suitability to remove quantization noise in EDA signals as well.

Here, we fixed a polynomial order to three and window size to five seconds and applied the filter to data from [4]. To compare this approach with traditional methods, we applied a moving average technique using the same window size, and its variant with applying a moving median beforehand as a classical approach to mitigate high-frequency spiky patterns. The results are depicted in Figure 5 comparing intact signals, and signals after smoothing by using hard low-pass FIR filter, S-G filter, moving-average, and moving-median-average techniques. The signal filtered by the S-G filter does not present oscillation as a result of quantization noise and is superior to moving-average and moving-median-average techniques in term of preserving the morphology of signals, especially the height of peaks.

The results above were obtained in a setting in which participants did not move. We further explored the effect of filters in another publicly available dataset from a study examining whether the degree of similarity of EDA recordings at both hands can be exploited as an objective method to detect movement artifacts in EDA signals [56]. EDA was recorded using the same device as in our study. Participants wore EdaMove 4 sensors at both hands and were instructed at known times to perform a certain movement using the right hand or arm while keeping the left stationary. The instructions included hand rotation, wrist bending, arm raising, thumb opposition of the fifth digit, electrode pressing, and no movement. High arousal affective sounds [57] were also evenly interspersed into the experiment to verify that fluctuations of the signal caused by mental processes (arousal) affect the signal in both hands in a similar way.

**Figure 6:**
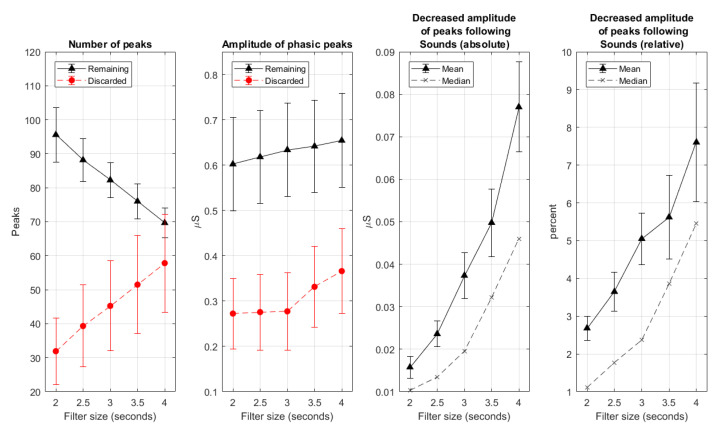
Effect of varying filter size from 2 to 4 s in steps of 0.5 s on the number and amplitude of phasic (remaining and discarded) peaks and on the attenuation of the amplitude of the peaks following emotional sounds (absolute values and relative values to the original peak heights in percent. Error bar represents SD/$\sqrt{N}$ where *N* = 12 participants.).

We investigated the effect of S-G filtering on data from the non-moving hand. CDA-Ledalab was applied to the original signal, and to the signal smoothed by S-G filters, where we varied the size of the S-G filter from 2 to 4 s in steps of 0.5 s. The effect of filter size was examined on the number of peaks, the amplitude of phasic peaks that remained and that were discarded after smoothing, and the reduction in size of peaks following emotional sounds (i.e., the “real” peaks, that were caused by arousing events), both in absolute value and relative value to the original peak height (in percentage). The results are plotted in Figure 6. As can be seen, the amplitude of discarded phasic peaks sharply increased when enlarging of window size beyond three seconds. Further expansion also resulted in abrupt increment of the attenuation of peak height following emotional sound, especially when considering median values. This suggests that the window size of three seconds might be a good compromise between clean signals and information loss.

In addition, we investigated the effect of S-G filtering (with a size of three seconds) on data from the moving hand. From visual inspection, we found that the artifacts due to movement have different characteristics than high-frequency quantization noise, especially in terms of morphology and peak amplitude. They were therefore not significantly impacted by the application of S-G filter.

**Figure 7:**
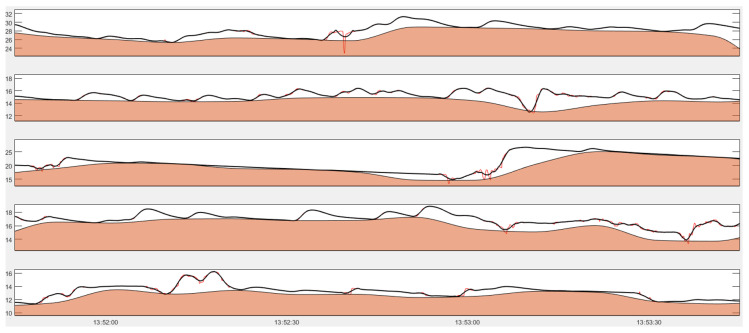
An example of EDA (in μS) of five participants in the same class. A red trace represents an original signal, a black trace represents the signal after applying S-G filter, and red shade represents the tonic level computed by CDA-Ledalab.

Given the capability of the S-G filter on reducing quantization noise while keeping psychophysiological signals intact, we applied a 3-second S-G filter with cubic polynomial order to the data that we recorded from school in the field. Figure 7 shows an example of EDA trace recorded from adolescent in school after applying S-G filter.

*Points of attention and recommendations for Section 3.2.2:* Device limitation in analog-to-digital conversion can lead to quantization noise, which can be mistaken as SCRs. To avoid false SCR detection, it is highly recommended to smooth EDA signals using an appropriate technique that can preserve important information including SCR occurrence and amplitude and can remove quantization noise. Our recommendation is to use S-G filtering.

#### 3.2.3. Movement Artifacts and Artifacts Due to Unknown Factors

In the previous sections, we discussed artifacts caused by loose sensors and quantization noise, and how to deal with them. Recording EDA in ambulatory settings using wearable sensors can result in a range of other artifacts such as those caused by pressure on the sensors due to body movements, which are often more difficult to recognize. Common practice is visual inspection. There were attempts to develop tools to automate the detection of artifacts. For example, Taylor and colleagues trained a machine-learning algorithm to detect movement artifacts in signals recorded by Q-sensor [58]. EDA features, such as amplitude, first and second derivative, and wavelet transform properties were extracted to train a support vector machine to discriminate between artifact and non-artifact data epochs, where ground truth valid and invalid data the labeling by two experts. The trained model was made available online allowing researchers to identify artifacts in their data using the offered web-based platform or downloaded code, named EDA-Explorer. Another approach was followed by Kleckner and colleagues [41]. They developed a rule-based approach for detecting artifacts in ambulatory EDA data recorded by the Q-sensor. Different parameters, such as temperature, SCR amplitude, absolute change (by more than 10 μS/s) of the SCL, and transitional period, were used to identify artifactual epochs, where the accuracy was confirmed by the agreement with ground truth from experts. Compared to EDA-Explorer, the rule-based approach marked fewer artifacts (24%) than the machine-learning-based approach (38%), using the same dataset.

The suitability of these tools to identify artifacts in data from other wearable EDA sensors has not been examined. The Q-sensor records EDA using a dry electrode at the wrist. Whether these tools are suitable to detect artifacts in signals recorded from a different site, such as the palm in EdaMove, is questionable. Differences in recording site can impact the characteristic of signals. Specifically, fingers and palm have a higher density of sweat glands compared to wrist causing differences in level of SCL, SCR, range, and sensitivity in response to arousing events [25]. EDA levels recorded from the wrist-type sensor were found to be typically around the lower bound of valid EDA range (0.05–60 μS) [7], making it hard to detect small EDA responses [59] and resulting in limited performance in detecting short mild stressors [60], especially when comparing with the performance of palm-based EDA device [61]. For a rule-based approach, it is necessary to establish specific criteria that match expert ratings [41], which may not generalize across signals captured from different devices, contexts, or study goals. Similarly, most effective features in EDA-Explorer for artifact classification are mean and shape (first and second derivative), which, as described above, can vary by the recording device, electrode type, measuring site, and external environment. It was found that when performing an experiment using the Empatica E4 wrist-worn EDA sensor in an air-conditioned room, EDA-Explorer detected artifacts for 2.77–17.38 % of the total data, which is considerably lower than 42.43–71.72% that was found when repeating the experiment in an outdoor environment which was hot (32–32 ${}^{\circ }$C) and humid, raising EDA level from 0–1.5 μS to 0–30 μS range [62]. This evidence questions the robustness of EDA-Explorer to variations in the range of the EDA signal, as can be encountered in real life.

**Figure 8:**
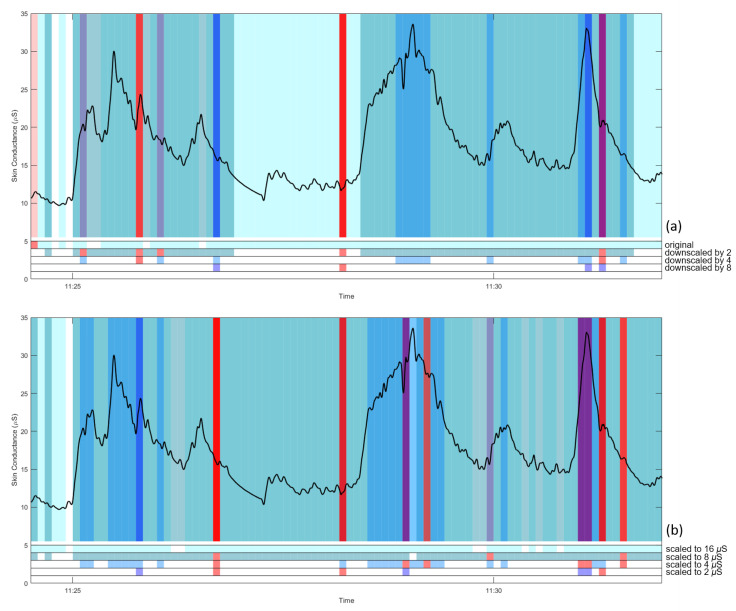
An example of artifactual (red) and questionable (blue-shade) segments as labeled by EDA-Explorer with the variation of the downscaling factors and rescaling to specific levels; (**a**) segments after downscaling (no scaling, 2, 4, 8) and (**b**) segments after rescaling to 16, 8, 4, 2 μS.

To further examine this, we used our collected data and probed the effect of different overall EDA levels. EDA signals, with removed signal-loss periods and S-G filtered as described before, were scaled down by dividing the original signal with a scaling factor of 2, 4, and 8. EDA-Explorer was employed to detect artifactual 5-s segments from the original and scaled data. Two different detection modes can be chosen: binary classification (artifactual vs. clean data) and multiclass classification (artifactual/questionable/clean data). Figure 8a shows an example EDA trace with artifactual and questionable segments as labeled by EDA-Explorer, for the different downscaling factors. The figure shows that the tool tends to mark high EDA values as artifactual, and low values as clean. It also shows a strong dependency on scaling factor, where the signal is considered almost completely clean when downscaled by a factor of 8. Similar results were obtained after downscaling the signal by shifting it down, such that mean values of 16, 8, 4, and 2 μS were obtained (Figure 8b).

Although downscaling can alleviate the problem of excessive artifact marking, it is difficult to define a suitable scaling factor. More importantly, the dependency of the result on overall EDA level is worrisome, since there is a large, natural variation in EDA level between individuals. The alternative available tool using the rule-based approach [41] also faces the difficulty of defining an appropriate threshold for artifact detection. For our data, we did not deploy an automatic tool to detect artifacts.

*Points of attention and recommendations for Section 3.2.3*: Neglecting signal characteristics when deploying an available automatic tool may lead to undesirable misinterpretation. Blindly using available tools for automatic artifact detection with default parameters may yield results that strongly, and incorrectly, depend on participant, recording device, and application. It remains necessary to validate the yielded artifact results by visual inspection, and adapt parameters for methods to better fit signals from other participants, devices and circumstances.

### 3.3. General Pattern

**Figure 9:**
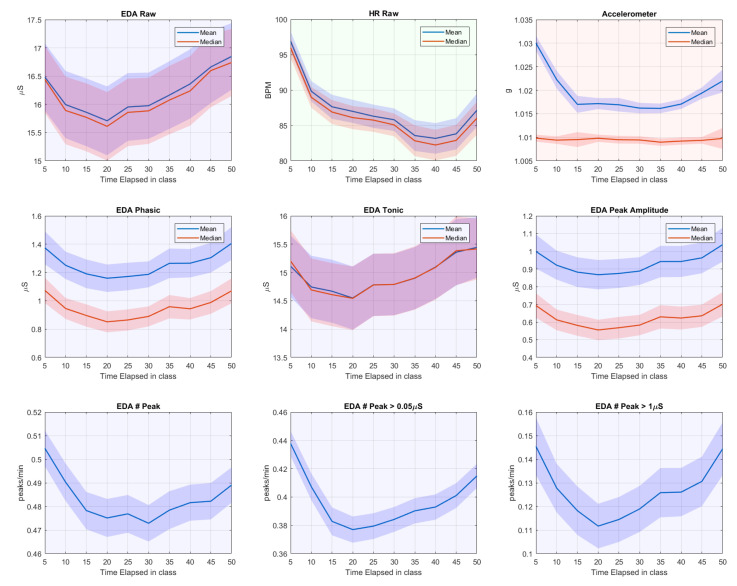
Features of EDA, HR, and accelerometer data over the course of 50-min class. A shade area represents SD/$\sqrt{N}$ where *N* = 85 participants for EDA and accelerometer and 80 participants for HR.

To explore the general pattern observed from students in school, we visualize how the varying EDA (after signal-loss period removal and S-G filtered signals) and HR features unfold over time during a class. Class duration–50 min for both schools–was divided into 10 slots of 5 min. For some rare classes that encompassed two consecutive 50-min periods, we concatenated the first 25 min from the first period and the last 25 min from the second period. For each 5-min slot, we averaged the features per participant and calculate the grand average over all participants. Figure 9 shows the results.

**Figure 10:**
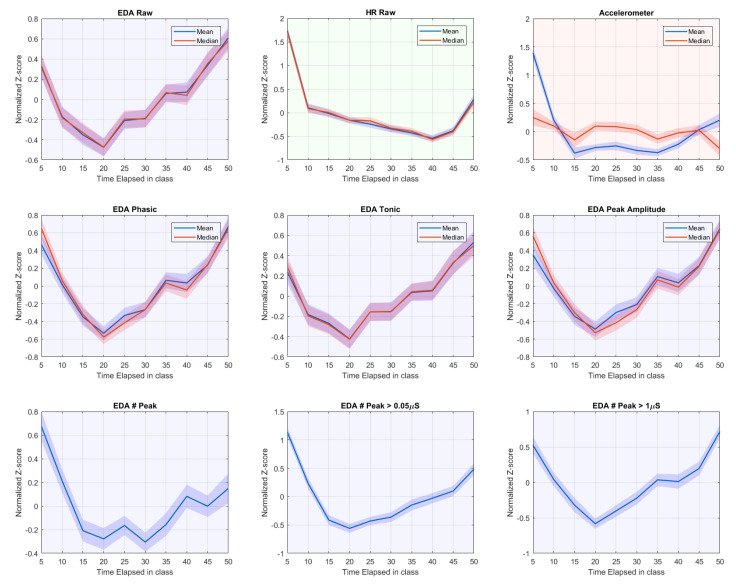
Normalized features of EDA, HR, and accelerometer data over the course of 50-min class. A shade area represents SD/$\sqrt{N}$ where *N* = 85 participants for EDA and accelerometer and 80 participants for HR.

To reduce variability between participants and the effect of individual long-term drift of SCL, we also calculated z-scores for each individual participant by normalizing features per class by mean and SD. The z-scores were then averaged across participants. Results of these normalized signals are shown in Figure 10.

It is conceivable that the students were settling down during the first 5–10 min. EDA and HR activities dropped precipitously, in good agreement with a prior study that observed physiological signals in school [23]. This period included much body movement, evidenced by accelerometer data and expected due to participants switching classrooms. Similarly, we observe the previously reported [23] rise in physiological signal and accelerometer-derived movement in the last ten min of class.

Figure 10 shows that, after the initial drop, EDA features start to increase from the 20th min of the class onward, which is before the movement-laden period at the end of the class. Meanwhile, HR continues to drop (consistent with previous findings [23]) until 10 min before the end when it starts rising again. Movement as indicated by accelerometer data also increases 10–15 min before the end. Together, these patterns suggest that while movement and HR are rather strictly coupled, there is no such strict coupling between movement and EDA, and the increase of EDA after 20 min may be due to mental processes, such as arousal caused by increased workload or engagement.

Note that, while the data above do not show a strong coupling between movement and EDA, we do expect some coupling, especially because movement heightens the chance of varying contact between skin and electrodes (see [56]). To further explore the relation between movement, and EDA signals and artifacts, we also calculated Spearman’s correlation between number of peaks with other features on a normalized scale. We found that the number of peaks with higher amplitude (>1 μS) was associated with EDA raw signal’s mean (rho = 0.523), phasic mean (rho = 0.873), and peak-amplitude mean (rho = 0.835), while the correlation with accelerometer data was low (rho = 0.261). However, when including the medium-size peaks (>0.05 μS), the correlation coefficients with the EDA mean, phasic mean, and peak-amplitude mean decreased to 0.364, 0.579, and 0.472, respectively, whereas the number of total peaks per minute correlated considerably more with the accelerometer data (rho = 0.403). When including all peaks (without minimum peak-height threshold), correlation with other EDA features was further lowered, while a stronger correlation with accelerometer data was obtained. Thus, the correlation between number of peaks with other EDA features is decreased by the presence of small peaks, while it boosts the correlation with accelerometer data. These results suggest that movement generates small- to medium-size peaked artifacts rather than high peaks. Therefore, the higher peaks should be more focused when exploring mental and cognitive states using EDA.

**Figure 11:**
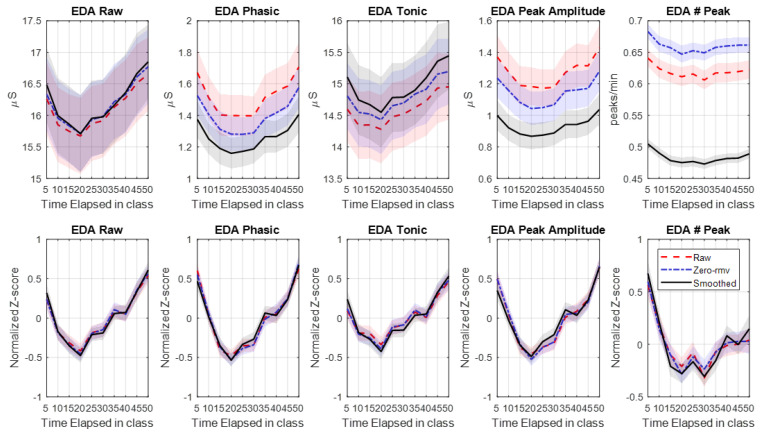
Comparison of (absolute and normalized) features calculated from raw EDA signals (Raw), signals after removing invalid portions (zero-rmv), and signals after applying the Savitzky–Golay (S-G) filter (smoothed) for all participants. A shade area represents SD/$\sqrt{N}$ where *N* = 85 participants.

To examine the benefits of removing signal-loss periods and data smoothing, we calculated features from signals in three different modes: original signals, signals after signal-loss period removal, and S-G filtered signals (and removal of signal-loss periods). Figure 11 shows that these preprocessing procedures affect overall mean values, but not the data pattern over time. This is also clear from the lack of effect of the procedures when using normalized values (bottom row Figure 11). This suggests that, at least when using EdaMove 4 in the classroom, and averaged across 85 participants, artifacts due to loose electrodes and quantization error do not necessarily affect feature patterns of interest.

**Figure 12:**
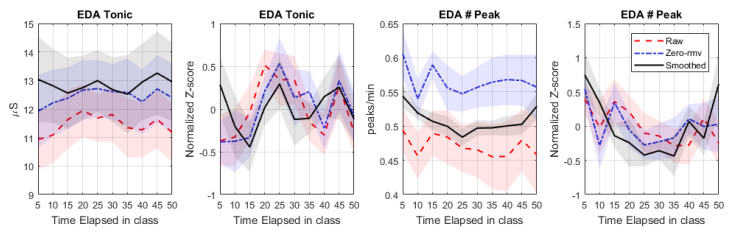
Comparison of (absolute and normalized) features calculated from raw EDA signals (Raw), signals after removing invalid portions (zero-rmv), and signals after applying the S-G filter (smoothed) for 11 participants whose signals were heavily contaminated by artifacts. A shade area represents SD/$\sqrt{N}$ where *N* = 11 participants.

As mentioned in Section 3.2.1, we found that the occurrence and duration of signal-loss period strongly varied across participants. Effects of preprocessing procedures may be substantial in participants with high data-loss. To further examine this, we focused on the 11 participants whose EDA signals were heavily contaminated by artifacts as explained in Section 3.2.1. Indeed, the effects of preprocessing procedures on features of interest are more salient as illustrated in Figure 12 (which shows only the features with apparent effects). Specifically, the dynamics of EDA features from this group are more consistent with the general U-shaped trend observed in Figure 11 after applying the S-G filter, suggesting that the proposed preprocessing aids in finding patterns of interest.

## 4. Discussion

With the present study, we reveal several important aspects regarding the usability and signal processing of physiological activity in real life. We collected electrodermal activity (EDA) and heart rate (HR) data during a school day using wearables in 86 adolescents. Measuring physiological signals in the field at school was found to be feasible with only minor interferences and a small amount of missing data. Our analyses of the data indicate that existing toolboxes in EDA research are helpful in signal preprocessing and analysis, but cannot be used blindly with default parameters. Neglecting differences between devices and measurement contexts would yield misleading results. In addition, objective detection of artifacts in ambulatory settings remains difficult and needs further elucidation. Thus, our general recommendation in ambulatory physiological measurement is that, after data retrieval, some primary guidelines should be followed. First, signal loss due to loose electrode should be investigated and invalid portions, including ultra-low signal, surrounding epochs and ultra-short epoch between invalid portions, should be discarded. Afterwards, quantization noise due to analog-to-digital conversion should be examined and, if existing, removed by applying a non-linear filter such as a Savitzky-Golay (S-G) filter. Next, artifacts should be removed with potential assistance of automatic tools and the awareness of possible confounding factors between mental state and movement artifacts. Finally, phasic components can be extracted from tonic level but the results should be verified with raw data. With proper consideration of the validity of signal processing and analysis techniques, ambulatory measurement of physiological signals using wearable sensors may lead to novel insights and applications.

In this study, EDA and HR data were collected at the palm and chest, respectively using wearable sensors. The assessment of such physiological activity during adolescents daily lives may be highly informative to examine mental state-related physiological arousal in everyday life and therefore to identify fine-grained processes to understand adolescent health and development. For this, precise and practical tools to measure this population of participants are strongly needed [63,64]. However, to date, the feasibility of using such tools among adolescents has rarely been examined. Regarding the usability of the device, our findings suggest that real-time data streaming may trigger the curiosity of adolescents, potentially leading to recording interference. Thus, devices that store data locally which could not be viewed or interfered by subjects may be preferable. The results of this research serve as the first evidence supporting the feasibility of measuring psychophysiological signals in-situ at school, where adolescents spend a large part of their day, and where controlling for (vigorous) physical activity is, at least in part, possible (e.g., by focusing on time spent in class).

We tested commonly used methods for signal preprocessing and decomposition on the acquired data. In particular, we aimed to provide insights into which settings may require additional attention for data collected with wearable EDA and HR sensors. Our study illustrates that applying available toolboxes while neglecting the characteristics of the specific signal under study can yield misleading results, such as falsely detected peaks. Specifically, we found that CDA-Ledalab overestimated the frequency of skin conductivity responses (SCRs), caused by quantization noise. Although the S-G filter can effectively deal with small, high frequency noise as induced by quantization error, it should be noted that the detection and manipulation of high-amplitude artifacts caused by electrode interference and body movement remain a hard problem given the S-G filter cannot perfectly remove artifacts with high amplitude. Future research should attempt to further address this issue. For example, the use of sparse recovery methods [65] and incorporation of accelerometer data could help to further deal with this issue, even though it was reported that the use of accelerometer data does not necessarily improve the quality of motion artifact removal schemes [66]. This may be partly due to the fact that at least EdaMove 4 signals seem quite robust to movements [56]. Deploying bilateral EDA difference to identify artifacts is an alternative method that has been recently proposed; this provides a context-free technique that might be a worthy option when EDA is recorded from two hands [56].

Our study suggests that the adoption of automatic tools should come with the realization of limited generalizability of tools across sensor types and situations (e.g., temperature and the presence of movement artifacts). Visual inspection of how the results of artifact detection methods compare to the raw signal is also important. Deploying tools with default values can lead to varying results depending on measuring context, device, and subjects. In our case, EDA-Explorer labeled most of our collected data as artifactual segments even after data smoothing, but the artifacts were no longer marked when data were downscaled. To our best knowledge, the sensitivity to the signal scale of EDA-Explorer has not been reported in the literature. Among 52 studies that deployed EDA-Explorer and explicitly cited the original article [58], 71% of the studies recorded EDA using the Empatica and 27% of them used the Q-sensor. These two devices measure EDA from the wrist, resulting in lower EDA level compared to those reported in the literature using palm and finger recording locations [41]. One of the 52 studies recorded EDA from the fingers using a Shimmer device. In this particular study, the overall EDA signal was also low (around 3 μS). The low level of EDA signals in these prior works might explain the lack of reports on problems with the validity of EDA-Explorer results. In general, if signal electrode sites and signal variation range are identical to the range in the Q-sensor or the Empatica device (dry-electrode wrist-worn device), EDA-Explorer or the rule-based method as described in [41] may be directly applicable. Otherwise, other methods to detect artifacts are required.

In a laboratory setting, it is often the case that approximately 10% of participants are considered to be ‘non-responders’, i.e., hypo-responsive to external stimuli [67]. However, our observations suggest that we cannot firmly regard any participant as a ‘non-responder’, but rather, that occurrences of hypo-responsive periods can be identified, although these are rare and last relatively short when compared with the whole recording period. In our case, such ultra-low EDA activity and scarce peaks mainly emerged during the first half of the class. Future research may test whether moving from laboratory to ambulatory settings results in finding less ‘non-responders’ or non-responding periods.

Overall findings from this study raise a number of important questions for future research and set the stage for future work on real-life physiological monitoring, both within the school environment as well as in other contexts. First, a critical future direction will be to incorporate incorporate ground-truth arousing events with a known timing, or expand the measurement to span several days increasing the chance to capture psychophysiological patterns. With specific regard to the school context, multi-day physiological measurement may reveal patterns potentially relevant to understand youth development of psychopathological symptoms, such as depression and anxiety [32,68]. It might also enable the possibility to examine the extent to which real-life physiological functioning is relevant to academic related-processes (e.g., academic motivation and achievement), and to investigate the mechanisms underlying the effects of social environmental experiences (e.g., social stress exposure) on youth development [69]. Moreover, it may be relevant to extend the analyses of physiological patterns in classroom settings by incorporating additional details. For instance, in socially anxious youth or youth exposed to a prior history of peer rejection, a class preceding a break may be associated with different physiological signals compared to a class preceding another class, due to the possibility of increased peer interactions. This type of more fine-grained analyses may contribute to a better understanding of individual-differences in physiological responses across contexts. Although the recording circumstances in this study were uncontrolled, we deliberately avoided selecting the classes that involved moderate to vigorous physical activities. Future work may also include this type of class, as physical activities are also associated with psychological/social and cognitive health indicators in school-aged children and youth [70], where EDA should be recorded from two hands of the participants to allow the investigation of motion artifacts in real-life recordings [56]. Furthermore, the analysis of physiological synchrony between students, which seems promising to study shared attention and emotion in real-life group environments, such as the classroom [17,71], could be promising endeavor.

A more general recommendation for future research is to explore how adolescents can eventually benefit from acquiring psychophysiological information; how we harness this information to support adolescents in their social and educational development. Examples range from improving educational material and optimal scheduling of lessons over the day, to detecting drops of attention in a student with attention disorders, to monitoring moments of stress in vulnerable students as an input to psychological treatment. Tied to this are safeguarding privacy and other ethical considerations.

In sum, the findings from this study support the feasibility to physiological functioning in real life. We identified a number of significant challenges in processing and interpreting data collected in a school setting among adolescents. The findings provide initial evidence of the usability of assessing physiological functioning among adolescents in a school setting, which provides valuable possibilities for future studies to implement wearables to examine real-life psychological processes at school. However, the recommendations and guidelines offered by this work may also guide future research assessing physiological functioning in different ambulatory settings among different samples.

## Figures and Tables

**Table 1:** Evaluation metrics for EDA removal settings.

Metric	Explanation	Normalization
Counts of jump artifacts (C1)	The absolute slope between an EDA datapoint and the following four datapoints (equivalent to 125 ms) were computed and averaged. Next, an arbitrary threshold was set as the minimum between the value of 2SD of the EDA signal and the value of 1.25 μS, equivalent to the change of 10 μS/s as recommended in [41]. We then counted the number of slopes that exceeded this threshold per participant in order to detect the frequency of “jump” artifacts.	normalization across settings, then averaging across participants
Mean of slope outliers (M1)	Mean and SD of the slopes in the previous metric were calculated. Next, we selected the slopes that deviated from the mean for more than 10 folds of SD and then computed the mean of these “outlier” slopes.	zero-mean adjustment, then averaging across participants, and then averaging across settings
Counts of short epochs (C2)	We counted the number of remaining EDA segments whose lengths were shorter than 10 s. The removal of the inter-loss period was expected to contribute to the decrement of this metric.	normalization across settings, then averaging across participants
Decreased entropy (M2)	The removal of unreliable EDA data should reduce the entropy of the signal. In each 10-second window, we computed the Shannon entropy based on probability distribution using the histogram technique [44]. Subsequently, we calculated the average of entropy per participant.	zero-mean adjustment, then averaging across participants, and then averaging across settings
Data loss (M3)	We calculated the ratio of loss data, due to the removal of the transitional and inter-loss period, to the total amount of data.	zero-mean adjustment, then averaging across participants, and then averaging across settings

## Abbreviations

The following abbreviations are used in this manuscript:

Ag/AgCl	silver/silver chloride
BPM	beats per minute
CDA	continuous decomposition analysis
DC	direct current
EDA	electrodermal activity
FIR	finite impulse response
HR	heart rate
SCL	skin conductance level
SCR	skin conductance response
SD	standard deviation
S-G	Savitzky–Golay
